# Livestreaming Technology and Online Child Sexual Exploitation and Abuse: A Scoping Review

**DOI:** 10.1177/15248380221147564

**Published:** 2023-02-02

**Authors:** Catharina Drejer, Michael A. Riegler, Pål Halvorsen, Miriam S. Johnson, Gunn Astrid Baugerud

**Affiliations:** 1 Oslo Metropolitan University, Norway; 2 School of Leadership and Theology, Oslo, Norway; 3 Simula Metropolitan Center for Digital Engineering, Oslo, Norway; 4 University of Tromsø, Norway

**Keywords:** livestreaming child sexual abuse, LSCSA, livestreaming, online child sexual exploitation and abuse, technology, CSEM, scoping review

## Abstract

Livestreaming of child sexual abuse (LSCSA) is an established form of online child sexual exploitation and abuse (OCSEA). However, only a limited body of research has examined this issue. The Covid-19 pandemic has accelerated internet use and user knowledge of livestreaming services emphasizing the importance of understanding this crime. In this scoping review, existing literature was brought together through an iterative search of eight databases containing peer-reviewed journal articles, as well as grey literature. Records were eligible for inclusion if the primary focus was on livestream technology and OCSEA, the child being defined as eighteen years or younger. Fourteen of the 2,218 records were selected. The data were charted and divided into four categories: victims, offenders, legislation, and technology. Limited research, differences in terminology, study design, and population inclusion criteria present a challenge to drawing general conclusions on the current state of LSCSA. The records show that victims are predominantly female. The average livestream offender was found to be older than the average online child sexual abuse offender. Therefore, it is unclear whether the findings are representative of the global population of livestream offenders. Furthermore, there appears to be a gap in what the records show on platforms and payment services used and current digital trends. The lack of a legal definition and privacy considerations pose a challenge to investigation, detection, and prosecution. The available data allow some insights into a potentially much larger issue.

## Introduction

Over the past three decades, the internet has evolved to become an essential tool to connect, sell, and inform, changing society as a whole, and with that, the scale and opportunities for committing internet-related crimes such as child abuse online (Wolak, 2004). The internet and mobile technology make possible the online viewing, distribution, and storing of child sexual exploitation material (CSEM) (Steel et al., 2020). Several offenses fit under the umbrella term of online child sexual exploitation and abuse (OCSEA), such as the production, dissemination, and possession of CSEM; online grooming; “sexting”; “sextortion”; revenge pornography; commercial sexual exploitation of children; exploitation through online prostitution; and the livestreaming of sexual abuse (Quayle, 2016). This review focuses specifically on the livestreaming of child sexual abuse (LSCSA), which is the real-time producing, broadcasting, and viewing of child sexual abuse and is related to sexual exploitation through prostitution, sexual performances, and producing CSEM (ECPAT, 2016). Unlike other forms of CSEM, such as images or videos, the LSCSA is the real-time production of child sexual abuse, which means that there is a potential to stop the abuse and protect the child while the abuse is occurring. Therefore, it is necessary to gain a better understanding of the issue and assess which tools are needed so that this abuse can be stopped or, in the best case restricted from happening at all. LSCSA is considered an established crime (European Financial Coalition against Commercial Sexual Exploitation of Children Online, 2015), and can refer to both commercial and non-commercial use, yet is perhaps most known for cases where the purpose is to exploit the child sexually for financial gain. A remote offender can view and direct the sexual activities online; in some cases, a facilitator arranges the abuse on site (European Financial Coalition against Commercial Sexual Exploitation of Children Online, 2015). The financial element leads LSCSA to be considered a form of commercial sexual exploitation of children, or child trafficking. Further, self-generated sexual content involving children and adolescents can also be a form of LSCSA. In these cases, there is not always a financial element present or a facilitator.

Several studies have looked at internet-facilitated sex offenses and commercial sexual exploitation with the internet as a facilitative tool or platform for the production, purchasing, and selling of CSEM or online live sexual content and the establishment of contact between the child and offender (Jonsson et al., 2014; Mitchell et al., 2011). However, limited studies have looked specifically at livestreaming technology as a service to facilitate the online sexual abuse and exploitation of children and adolescents, which is notable, as the technology has been used by offenders for at least two decades. In 2001, it was reported that web cameras were used to facilitate child sexual exploitation and abuse through live transmission (Hughes, 2001; 2002). Mitchell et al. (2011) state that 5% of offenders arrested in 2006 for internet-facilitated commercial sexual exploitation of children in the United States had watched live sexual activity containing minors using the internet. In 2008, a study by Shannon examined the extent and character of online sexual grooming in Sweden and identified web cameras as a tool used by offenders. Images were saved and used as blackmail, also known as sextortion, to obtain more images or to coerce the child to provide live sexual content using a web camera (Shannon, 2008). Multiple court cases from the Netherlands show that in 2009 web cameras were used by adults to extort young children to perform sexual activities (Koops, 2009). These studies have included the component of livestreaming in their investigations and analysis, often referring to the web camera as a tool. However, studies with empirical data on LSCSA with a web camera or other devices are limited (Koops et al., 2018).

### Livestreaming Technology

Livestreaming is the term for operations or technology that allows recording and broadcasting over the internet in real time (Hamidouche et al., 2022). The main technical steps in a livestreaming pipeline are compression, encoding, segmentation, distribution, caching, decoding, and video playback. Current solutions are argued to be both cost-effective and easy to use without a need for complex separate setups (Fecheyr-Lippens, 2010). Almost every social media platform allows person-to-person livestreaming in one way or another (Facebook live, TikTok live). This is also true for (1) message apps like WhatsApp, Discord, and Facebook Messenger; (2) specific services focused on livestreaming like Twitch and Omegle; and (3) video call software that has become common during the Covid-19 pandemic such as Microsoft Teams, Zoom, and Google Meet. The terms web cams or web cameras were used early in the video streaming age, but now, however, technology enabling livestreaming includes anything with a camera and a network connection, ranging from high-end professional cameras to computer cameras, mobile phones, and other internet of things devices such as glasses, watches, and drones. Finally, it is interesting to note that from a user perspective, livestreaming has not changed significantly in recent years, besides better video quality, a better experience with no delays, and easier accessibility.

### Livestreaming of Child Sexual Abuse

Over the last few years, an increase in LSCSA has been noted (Virtual Global Taskforce, 2019). The COVID-19 pandemic has accelerated the use of digital technology worldwide, and Europol (2020b) found that LSCSA has increased and become more prevalent during the pandemic. Moreover, they note that in cases of OCSEA, it was expected that LSCSA would increase while travel was restricted and borders closed (Europol, 2020b). The Canadian National Child Exploitation Crime Centre recently stated that “with the pandemic we have seen an uptick in livestreaming with overseas victims” (Somos, 2022). In the Philippines, which is described as the global epicenter of the livestream sexual abuse trade (Brown, 2016), a significant increase in child sexual abuse cases were reported during the first months of Covid-19 compared to the year before (Pulta, 2020). In addition, the amount of self-generated sexual content featuring children enabled by livestream services has been rising (Internet Watch Foundation, 2020). On the demand side, Europol (2020a) found a significant increase of interest in video captures (“cappers”) of LSCSA on dark web forums.

Considering this increase in the use of livestreaming technology, accelerated by the Covid-19 pandemic, and the corresponding rise in cases of OCSEA, it is critical to understand better the unique characteristics of LSCSA. In this scoping review, we present the relevant literature and examine the range and nature of the evidence on LSCSA by answering the following research question: *What are the characteristics of LSCSA; who are the victims and offenders; and what are the enabling technologies?* Our aim is that these findings will present an overview of the evidence and address the gap in the literature on LSCSA which can direct future research.

## Method

To answer the research question a scoping review was conducted. Scoping reviews are used to scope the extent, range, and nature of the evidence and identify research gaps while following a systematic approach (Tricco et al., 2018). Furthermore, scoping reviews lend themselves especially well to emerging fields and for organizing and summarizing heterogeneous evidence without evaluating the quality of the studies. This scoping review explores the literature and brings together the emerging evidence on LSCSA. The Joanna Briggs Institute (JBI) framework for scoping reviews and the PRISMA Extension for Scoping Reviews checklist have been utilized to ensure the quality and transparency of the review conducted offering guidance in developing a research question, inclusion, and exclusion criteria, extracting, and charting the results, and discussing these with their implications for future work (Peters et al., 2020; Tricco et al., 2018).

### Search Strategy

We used the three-step search method suggested by the JBI to ensure a comprehensive search strategy. First, a wide selection of databases was selected to gather the data and to ensure adequate coverage of the literature to minimize publication bias. Records were identified using iterative searches of ACM Digital Libraries, EBSCOhost Academic Search Complete, Google Scholar, IEEE Explore, ProQuest, PsychINFO, Springer, and Web of Science in June 2021. Search strategies and terminology were modified as necessary for each database. Second, we searched for keywords and used three separate elements: child, abuse, and livestreaming technology. Then we added synonyms. These three elements were then combined in the search, which yielded results. Table 1 shows an example of the electronic search strategy for the PsychINFO database. Third, the references of the included articles were screened to retrieve documents and studies both in and outside of scholarly literature. Records that were eligible for inclusion had to meet several criteria. (1) A primary focus on the phenomena of interest: livestream technology and OCSEA. Due to the nature of a scoping review, the criteria were not given additional descriptors as this would have narrowed the scope too much. Records that did not focus on live streaming as a means for online child sexual abuse were excluded. As an example, some nongovernmental reports that were selected had a focus on online child sexual abuse in the Philippines which is known to have many cases of LCSA; however, because there was no specific focus on the livestreaming element of child sexual abuse, these were excluded. (2) The victim was defined as a child or adolescent 18 years or younger according to the Convention on the Rights of the Child (United Nations, 1989). For example, one study included victims who surpassed the age of eighteen due to recording practices of law enforcement. Even though this was the case in a limited number of cases, the study was excluded. For offenders, no particular age was set as a criterion. (3) Newspaper articles, bachelor and master theses were excluded, and all other documents were included. (4) The included records had to be written in Danish, English, Norwegian, or Swedish. There were no criteria set on methodology, or year of publication.

**Table 1.** Electronic Search Strategy APA PsycInfo.

Query	Search Terms Used	Results
1	Child behavior/	450
2	Child psychiatry/	6,923
3	Child attitudes/	7,432
4	Child psychology/	4,398
5	Child neglect/	4,363
6	Child health/	218
7	(child* or baby or babies or infant* or newborn* or toddler* or youth* or young* or preteen* or pre-teen* or Teen* or adolescent* or kid* or pre-pub* or prepub* or pre pub* or pubescent* or pubert* or post-pub* or post pub* or postpub* or peer* or juvenil* or underage* or minor* or boy* or girl*).mp.	1,510,712
8	Query no. 1 or 2 or 3 or 4 or 5 or 6 or 7	1,510,712
9	Exp child abuse/	31,026
10	Sexual abuse/ or incest/ or exp rape/	28,801
11	Human trafficking/ or sex work/	4,917
12	Pornography/	2,367
13	Exp cybersex/	556
14	Sex offenses/	11,057
15	(porn* or exploitat* or (sex* adj2 (abuse* or tourism* or traffick* or assault*)) or cyber traffick* or prostitution or molest* or cybersex* or erotic* or rape* or incest* or virtual* abuse*).mp.	66,959
16	Query no. 9 or 10 or 11 or 12 or 13 or 14 or 15	90,068
17	Streaming technology/	22
18	Digital technology/ or digital video/ or internet/	32,370
19	(stream* or (live adj2 (stream* or video* or distance or broadcast* or online* or footage or webcast* or image* or feed* or transmission* or video communication*)) or videocast* or on demand or webcam* or web cam* or web-cam* or ((indecent or inappropriate) adj2 video*) or (online adj2 child adj2 sex*)).mp.	18,871
20	Query no. 17 or 18 or 19	50,727
21	Query no. 8 and 16 and 20	792
22	Limit Query no. 21 to (danish or english or norwegian or swedish)	759

### Protocol and Extracting Results

**Figure 1.**
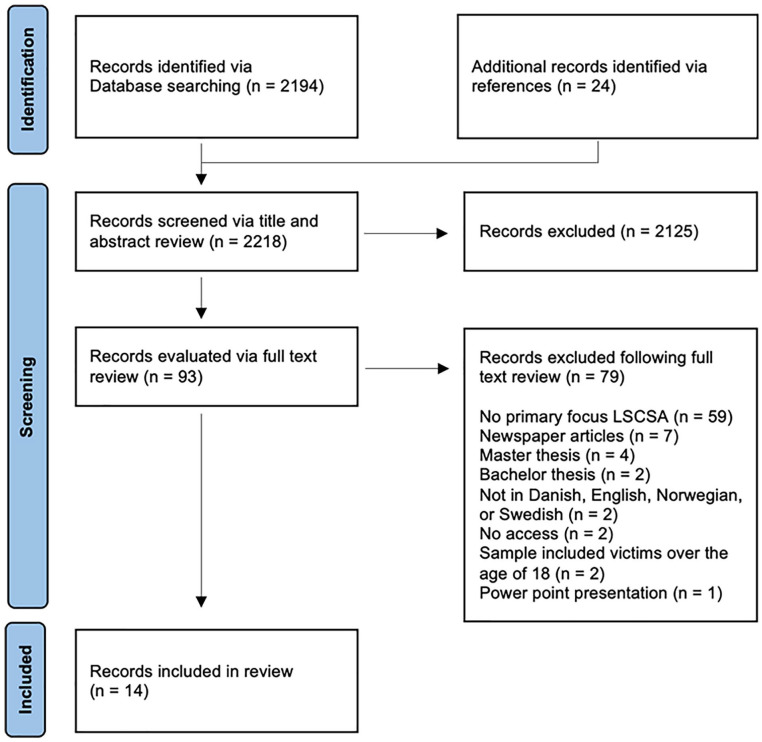
PRISMA flowchart summarizing review methodology.

A protocol was developed based on the Reporting Items for Systematic Reviews and Meta-Analyses Extension for Scoping Reviews (PRISMA-ScR) Checklist (Tricco et al., 2018). This protocol functioned as a guide to make clear and consistent decisions as a team and was uploaded in the Open Science Framework under DOI 10.17605/OSF.IO/G6EUB. The titles and abstracts of the records (*n* = 2,218) were screened by at least two independent researchers. Similarly, two independent researchers screened the full texts (*n* = 93). Data were extracted, and summaries were made of each of the records. Where there were conflicts, a discussion was held with the members of the team to decide on either inclusion or exclusion. Seventy-nine records were excluded because they did not meet the two criteria of having a primary focus on livestreaming of online child sexual abuse and the child being defined as 18 or younger. A total of 14 records were identified for inclusion, see Figure 1 for the PRISMA flowchart. A data-charting form was jointly developed by the reviewers to determine which variables to extract. These included the author(s), year of publication, location, language, aims of the study or record and main area of focus, methodology, important findings, technological definitions, terms used throughout the record, and policy recommendations. The results were identified and organized by content and listed in Table 2, which shows the dominant areas of research in terms of the research topic, method, and geographical location of the data. Four primary categories were identified: victims, offenders, legislation, and technology (technical challenges and solutions concerning livestreaming). There are substantial overlaps within these records, as they often touch on several of the categories. Using the four mentioned categories provided a framework to organize these findings in a systematic way.

**Table 2.** Overview of Included Records.

Record	Method	*N*	Primary Topics Discussed				Main Focus Philippines
			Victim	Offender	Legislation	Technology	
Terre des Hommes (2013)	(1) In-depth interviews with victims, their families and community members. (2) A combination of a semi-structured interview and a structured questionnaire.	44 cases with 65 respondents	X	X			X
		48+ respondents					
Terre des Hommes (2014)	Online field research interacting with 20,172 offenders on 19 chat rooms over a ten-week period.	1000 identified offenders		X	X		
Açar (2017)	Discussion of current and futuristic methods of detection.	N/A			X	X	
Varrella (2017)	Discussion of definition, causes, consequences, and the existing legislative international and national frameworks.	N/A			X		X
Internet Watch Foundation (2018)	Content analysis of images from a sample of captures of livestreamed child sexual abuse available online.	2,082 image and video captures	X				
Horsman (2018a)	Examination of the forensic procedures required reconstruction of web-browser cached video data from YouTube and Facebook streaming services.	N/A				X	
Horsman (2018b)	Investigation of the challenges posed by the livestreaming platform “Periscope” in order to reconstruct cached data.	N/A			X	X	
Horsman (2019)	An analysis of video stream reconstruction using web-browser cache data from the Twitch, Youtube, Mixer, Ustream, Smashcast, and Younow livestream services.	N/A				X	
Dushi (2019)	A comparative legal analysis between Italy, and England & Wales.	N/A			X		
Dushi (2020)	Discussion on the current existing legislation and its implications on the crime of livestreaming child sexual abuse.	N/A			X		
Brown et al. (2020) *	Analysis of financial transactions of Australian-based individuals to known livestreaming facilitators in the Philippines. (Based on the same data source as used in Cubitt et al., 2021).	256 individuals and 2,714 transactions		X			X
Cubitt et al. (2021) *	Analysis of financial transactions of Australian-based individuals, using machine learning, to identify characteristics of prolific livestream offenders. (Based on the same data source as used in Brown et al., 2020).	207 individuals		X			X
Napier et al. (2021a) *	Analysis of chat logs from offenders who watched and directed the sexual abuse of children via livestream. (Based on the same data as used in Napier et al., 2021b).	7 offenders and 74 victims	X	X			X
Napier et al. (2021b) *	Analysis of chat logs of offenders committing child sexual abuse livestreaming offences. (Based on the same data as used in Napier et al., 2021a).	7 offenders, 145 offences and 74 victims		X			X

* The four studies are conducted at the Australian Institute of Criminology and for some the same dataset is used.

## Findings

The 14 records included eight peer-reviewed articles, a PhD study, a book chapter, and four reports by nongovernmental organizations (Table 2). In the section below these findings are categorized into the four categories of interest: victims, offenders, legislation, and technology to discuss the characteristics of LSCSA. It is important to note that most of the data in the victim and offender categories are based on cases from the Philippines. Further, four of the empirical studies are interrelated (Brown et al, 2020; Cubitt et al., 2021: Napier et al., 2021a, 2021b). For a summary of the most critical findings, see Table 3.

**Table 3.** Critical Findings.

	Findings
Victims	* The victims are mostly female. *Victims are manipulated into participating rather than being forced because they know their abuser. * Victims also contact offenders themselves with the motivation to earn money. *Victims report feeling shame, fear, and embarrassment and suffer from high levels of psychosocial distress. *The material of the abuse is spread which makes for revictimization.
Offender	* The offender is considered to be a facilitator of the abuse and/or the person viewing or ordering the livestream. * The livestreaming offender is generally older than other online offenders. * Some offences were opportunistic in nature. * The offender used well-established platforms for both making contact and streaming. * Offenders usually payed with well-established money transferring systems.
Legislation	* Livestreaming of child sexual abuse is not a stand-alone offence and has no legal definition which creates challenges in conviction of the crime. * The lack of a common definition also challenges the scientific work.
Technology	* Detection is difficult. * Privacy regulations make the creation of regulations and laws challenging. * There might be a big gap between the services that are known to be used and the number of services that are used (Table A1 in the Supplemental Appendix). * Livestreaming is built in almost every social interaction platform existing.
Overall/general	* There is lack of consistency in definitions and operationalizations across studies in the literature. This challenges the comparison and generalization of the findings and applicability must be carefully examined.

*Note*. Reviewed 14 records. The findings were divided into four different categories: victim, offender, legislation, and technology.

### Victims

Most of the records included in the review describe the characteristics of the victim and the process of victimization. Only one record presented some of the psychological effects live streaming of child sexual abuse has on its victims. These are all key components to a better understanding of the LSCSA. The ages of the victims ranged from 7 to 17, and the victims were predominantly female (Internet Watch Foundation, 2018; Napier et al., 2021a; Terre des Hommes, 2013). Three models of operation have been identified in the LSCSA (Terre des Hommes, 2013). However, it is important to note that these models of operation are based only on data from the Philippines. (1) “The individual operation” in which a child voluntarily performs sexual acts on camera for foreign viewers in exchange for money. Note that this child is considered a victim and that it is often not clear to what extent the child is coerced. (2) “Family-run operations” that most often involve either parents or family members coercing the child to perform sexual acts on camera in exchange for money. (3) “Cybersex dens” in which several children are either hired or trafficked and kept against their will in a location to perform sexual acts on camera (Terre des Hommes, 2013).

#### Victimization

Victimization refers to the process of becoming a victim of LSCSA. Although the Philippines is considered the hotspot of the LSCSA (Brown, 2016), the records show that victims are also located in other parts of the world. Out of 74 victims identified in Napier et al. (2021a), 43 were from the Philippines, while two were located in the United Kingdom, two in China, and one victim was in Thailand. Of the remaining 26 victims, the location was unknown. The Australian offenders also attempted to establish online contact and groom children from Australia, Indonesia, Vietnam, Japan, and Namibia (Napier et al., 2021a). Furthermore, the Internet Watch Foundation (2018) found that victims are mostly white females from relatively wealthy Western backgrounds. This indicates that victimization takes place globally.

Family members, peers, and community members have a significant role in persuading or manipulating a child into participating in LSCSA (Terre des Hommes, 2013). These people related to the child either facilitate or participate in the abuse. The facilitator was often found to be a relative of the victim, most commonly their mother or sister (Napier et al., 2021b). Due to this type of family involvement, the child is not necessarily physically forced to participate, but simply obeys the parent or is manipulated into participating. This is supported by earlier findings that most children who appear in CSEM have not been physically forced to participate. Instead, they were often manipulated into participating because they knew the person facilitating the abuse (Worthley & Smallbone, 2006). Almost half of the victims have family members who are themselves involved in LSCSA or are aware of the child’s involvement in sexual activities on the web camera, which suggests a degree of normalization within the community (Terre des Hommes, 2013). Poverty is considered one of the main driving forces behind the LSCSA (Brown, 2016; Hernandez et al., 2018; Varrella, 2017). Most of the studies in this review are based on data from the Philippines, where poverty levels are high—about 24% were living below the poverty threshold during the first half of 2021 (Philippine Statistics Authority, 2021). LSCSA is perceived as relatively harmless because in some cases there is the absence of contact abuse. Moreover, it is considered an easy way to provide income for the family (Terre des Hommes, 2013; Varrella, 2017). In addition, children in these environments are often expected to help provide an income for the family, especially when there is a lack of basic needs (UNICEF, 2021). There are also cases where children themselves reach out to foreigners, without their parents’ knowledge. In these cases, the child is either pressured or lured by peers and learns to establish contact with a foreigner through their network of friends. The motivation is often to provide an income for their family or themselves (Terre des Hommes, 2013). Two common methods are identified for how offenders have targeted their victims: (1) by contacting women and teenagers online to establish relationships, whereafter the offender would request access to younger children or be offered access; and (2) by establishing relationships with families while visiting the Philippines for work. Upon return, they would request CSEM and LSCSA (Napier et al., 2021b; Terre des Hommes, 2013). Even though this type of abuse often includes a facilitating person, in two-thirds of the analyzed cases, offenders communicated directly with their victims (Napier et al., 2021b). Sometimes grooming tactics were used, such as “providing compliments,” “using romantic language,” and “asking inappropriate or personal questions” (Napier et al., 2021b, p.12; Terre des Hommes, 2013). Offenders would build relationships with the facilitator and victims by promising education for the child, payment of tuition fees, or payment for other material needs in return for participation in LSCSA (Terre des Hommes, 2013).

#### Psychosocial consequences

Terre des Hommes (2013) interviewed 44 children from the Philippines who were involved in livestreamed sexual abuse or had been rescued. Regarding the psychosocial consequences, victims reported feelings of shame, fear, and embarrassment. The victims suffered from high levels of psychosocial distress such as traumatic sexualization, betrayal, social stigmatization, and powerlessness as defined by the traumagenic dynamics model by Browne and Finkelhor (1986) for understanding the trauma of sexual abuse. The traumatic effects on the victims were severe due to the involvement of their parents and included feelings of confusion and conflict of loyalty (Terre des Hommes, 2013). This presents challenges for the disclosure of the crime and the child’s psychological recovery, as they will have to learn that their parent has committed a crime and might be incarcerated for the offence (Terre des Hommes 2013). A common finding throughout the literature is that children are less likely to disclose sexual abuse when the victim is close to the offender (Hershkowitz et al., 2014) and when motivational factors such as negative emotions and concerns of legal consequences to family members are present (Malloy et al., 2011). However, these experiences are not necessarily unique to the LSCSA since, for other types of online child sexual abuse, parents were also found to be facilitators and offenders (Mitchell et al., 2011). Family reintegration is challenging due to the risk of revictimization and the fact that family and community members either actively took part in or tolerated the crime and failed to protect the child. Another type of revictimization, that has been noted for OCSEA, is the online spreading of CSEM. Once an image is online, the “image exists out of the subject’s control for the remainder of his or her life” (Quayle & Sinclair, 2012, p. 15). This also applies to other content on the internet such as videos. Although it is argued that livestreaming leaves no trace due to the live transmission component, the records suggest that screen captures or cappers are taken, redistributed, and stored just as with images and videos (Europol, 2020a; Internet Watch Foundation, 2018; Varrella, 2017). Interestingly, the Internet Watch Foundation (2018) did not find any captures of livestreaming made available to remote buyers in their search of the surface web. This could support the claim by Varrella (2017) that the material can be disseminated through the dark web or other private networks (Internet Watch Foundation, 2018). Cappers from livestreamed abuse are often uploaded to cyberlocker sites. These sites help to commercialize the provision of content by paying the uploader per download from the content-providing cyberlocker. The links to these cyberlockers are distributed within dedicated online forums that often use images of child sexual abuse or captures to promote downloads to those interested in CSEM (Internet Watch Foundation, 2018). Some of the content when located can be removed from an online location. However, it cannot be removed entirely, and the victim must live with this reality.

### Offenders

This section presents the findings regarding offenders of LSCSA, including offender characteristics, and the enabling platforms. There is a distinction between different types of offenders, and the records use different terminology in their descriptions. The person who views, directs, and pays for the LSCSA is often referred to as either the “customer,” “perpetrator,” “client,” “consumer,” “offender,” or “predator.” If there are people “on-site” facilitating the exploitation and abuse, they are referred to as the “facilitator,” “operator,” “middleman,” “pimp,” or “trafficker.” However, not all cases have a facilitator, for example when the abuse is self-generated. Furthermore, the facilitator can also be the offender (Napier et al., 2021b). These differences in terminology make it challenging to compare the data.

#### Characteristics

Regarding the characteristics, Cubitt et al. (2021) analyzed the age of offenders of LSCSA and found that ages ranged from 20 to 76 years, and the average age of the offender was 52 years. Other studies have shown a range of between 50 and 60 years and 42 and 72 years of age (Brown et al., 2020; Napier et al., 2021a). These studies have used the same data source to some extent, and it is therefore unclear if there is any overlap in their findings. A distinction was made between low-volume and high-volume offenders (Cubitt et al., 2021). High-volume offenders were the 10% of the data sample with the highest number of transactions. The study used machine learning to predict the characteristics of prolific LSCSA offenders and found that offenders who made more than one transaction showed a decline in time between transactions, which was confirmed by Brown et al. (2020). With the increase in the frequency of transactions, the severity of the offending also increased (Brown et al., 2020). High-volume offenders appear to make low-value transactions, but more frequently. It was unlikely that an offender would spend more than 250 USD in a single transaction. The study’s authors raise the question of whether this is a strategy to avoid detection. In addition, the study looked at the criminal records of these offenders and found that a history of low-harm offending was common. Prior sex offences were not a significant predictor of prolific livestreaming (Cubitt et al., 2021). Further, a number of the offences were “opportunistic in nature” (Napier et al., 2021b, p.14); for example, the offender was offered an opportunity to watch child sexual abuse while initially paying for adult women to perform sexual acts.

#### Technical platforms and payment

Most offences have been committed on the surface web using computers (personal or public), laptops, tablets, and mobile phones (Napier et al., 2021a; Terre des Hommes, 2013). Furthermore, the records find that well-established platforms are used to initiate contact via chat and to facilitate and view LSCSA: Facebook (Messenger), Yahoo! Messenger, text messages, Skype, and Viber (Napier et al., 2021a; Terre des Hommes, 2013). Negotiation of the price and method of payment was common and established prior to streaming, typically using online chat (Napier et al., 2021a). Various factors determine the price: the victim’s age, the extremity of the abuse, the length of the stream, and the number of children involved (Terre des Hommes, 2013; Varrella, 2017).

The Philippines has a well-established money-transferring system, and remittance services are a common way to send and receive money (Varrella, 2017). The most popular services are Western Union, WorldRemit, Remitly, and PayPal which provide more anonymity (Napier et al., 2021a; Terre des Hommes, 2014). The prices for LSCSA per show are generally not very high, ranging from $10 to $50 USD (Terre des Hommes, 2013; Varrella, 2017). However, in some cases the amount was higher but not exceeding $404 AUD which is the equivalent to around $250 USD (Napier et al., 2021a). Higher payments were an indication of the personal involvement of the offender in the children's lives.

### Legislation and Technology

It is argued that existing laws do not adequately address the specific offence of livestreaming (Dushi, 2020). LSCSA is not a stand-alone offence, and criminal courts have used other provisions to criminalize the LSCSA, such as child pornography, child prostitution, and rape (Dushi, 2020). To adequately protect children in the online environment, legislation should “enable to identify, locate, investigate, and prosecute online offenders” of LSCSA (Dushi, 2020, p.220). Although there are national laws and international instruments^1^ addressing OCSEA, they fail to sufficiently address the criminal acts of LSCSA (Dushi, 2020). However, they do present a framework for creating comprehensive new legislation addressing OCSEA more explicitly (Dushi, 2019, 2020). When national laws and international instruments are not harmonized, legal loopholes can appear. The absence of harmonized legislation can also lead countries, judges, and courts to interpret the same instruments differently (Dushi, 2019, 2020). This makes cross-border collaboration challenging and affects scientific research, as many definitions are used because of the lack of agreement upon legal terms. In the literature, LSCSA is also referred to as “live online child sexual abuse,” or “child sexual abuse to order” (ECPAT, 2016), “live distant child abuse” (Europol, 2015), “pay-per-view,” “on-demand child sexual abuse” (Ramiro et al., 2019), “webcam child sex tourism” (Terre des Hommes, 2013), or “webcam child prostitution” (Açar, 2017).

In addition to these legal challenges, there are challenges related to creating and using technical solutions to investigate and detect LSCSA as well. Terre des Hommes (2014) argued for adopting proactive law enforcement policies, creating policies and legislation that enable law enforcement agencies to patrol public online spaces. Their study used an automated chatbot, an artificial intelligence feature that simulates human conversation through, for example, text-chat. In this case, the chatbot was a 10-year-old Filipino girl called Sweetie. The chats identified individuals seeking to engage in LSCSA in 19 chatrooms. Although the researchers interacted with 20,172 offenders and identified 1,000 offenders from 71 different countries, using chatbots to perform this type of investigative work is legally and ethically challenging. Açar (2017) examined different technologies that can be used to detect LSCSA. Fully automated chatbots such as the above-mentioned Sweetie 2.0, utilizing big data analysis of meta-data and analysis of content data provided by Voice-over-IP companies, to avoid having to rely on human reporting, are discussed. This, however, is intrusive, violates privacy, and will require changes of laws and rules. The technical difficulties are solvable, but the real challenges lie in legal and socio-psychological complications.

From a technical perspective, it is very difficult or even impossible to detect and observe livestreamed abuse from the outside (usually such a livestream is performed in a secured environment, an encrypted connection, between two or more parties). However, Horsman (2018b) presents a method for investigating live streams in Periscope (Twitter’s livestream service). Live analysis is necessary to identify abusers and is done with full internet access and access to the service. Thus, for live access, two different sides exist: the victims and the offenders. For the live analysis, access to the account and perhaps even the device is needed to extract information. If only the victim’s side is known, cooperation from the provider is necessary to determine if the offender made a fake profile. Thus, with only the victim’s perspective and information the victim can provide, enforcement of a sexual abuse offence involving a livestream video is practically difficult. It is challenging to find concrete evidence of the usage of a live sexual abuse case. The information that can be extracted might also differ between different devices. Live access and live analysis are needed to obtain information that can be used legislatively. This might be impossible depending on the devices used. The Horsman study was performed with Periscope only, but these findings are likely valid for all livestream services. One way to investigate livestreamed videos is by looking at cached content on the devices of the victim or offender as discussed by Horsman (2018a). Reconstructing videos after they are streamed is one way to investigate live sexual abuse. Rebuilding from cached video stream data is necessary. Cached metadata for video stream files are needed to determine the order. If this is not available, the task becomes unfeasible and perhaps even impossible because of the many different files. Viewing recorded livestreaming leads to cached content from all platforms investigated by Horsman (2019) in a follow-up study. However, livestream broadcasts are only cached for four of six services (i.e., Twitch and YouNow do not support this). In general, further work is needed to obtain a better understanding of the technical challenges and the differences between the platforms. Forensics is at the beginning of investigating livestreams and the different platforms, and it is challenging to reconstruct and find content. Browsing history and cached content need to be combined to allow proper investigation. Content providers could help make the task easier, but governmental rules or laws will be needed to achieve this. Machine learning might be a useful tool in making the task more feasible.

On the other hand, despite the possibility of recreating data from web-browser caches and finding usernames in the metadata (Horsman, 2018a, 2018b), application providers are also building support in their systems for a user to stay “private.” For example, a technical issue is that many modern browsers support what is called “private mode” where all browsing history and content is removed as soon as the window closes. Thus, all the evidence will automatically be deleted, or not be accessible at all as in many mobile apps. This presents a great challenge in making legislation, rules, and laws to regulate this. As noted above, an efficient detection of online sexual child exploitation and abuse may require full access to the server, network, and client systems, but this goes against the principles of enabling full privacy.

## Discussion

This scoping review identified 14 records. The included records had a primary focus on livestreaming of online child sexual abuse, the child being defined as 18 years or younger. This study included peer-reviewed journal articles as well as grey literature. No rating of the quality of evidence is provided but an overview of the evidence is presented. No general conclusions can be drawn about the current state of LSCSA on an aggregated, global level. Arguably, most studies are more informative when looking only at the Philippines. A major limitation is that there is no agreed-upon legal term for the LSCSA. Further, the variety of study designs, research questions, and variable operationalization such as distinctions between facilitators and offenders, made it challenging to retrieve relevant data and for future studies to compare these data. In addition, the study samples were relatively small making the findings non-generalizable.

### Victims and Offenders

Gender and cultural dimensions may influence what is observed in the data. For instance, most victims of LSCSA represented in the data were female. This could either reflect that victims of LSCSA are predominantly female or that male victims are underrepresented in the data. In fact, studies on child sexual abuse suggest that male victims tend to delay the disclosure of sexual abuse which could explain the observed numbers (Gagnier & Collin-Vézina, 2016). This is therefore an important area of further study. Conducting research that is inclusive and gender sensitive will affect the identification, treatment, and support of the victim.

The data on the LSCSA primarily originate from the Philippines. The Philippines is referred to as “the global epicentre of the livestream sexual abuse trade” (Brown, 2016, p.1), as most of their cases of online child sexual abuse are enabled through livestream services (Europol, 2019; International Justice Mission, 2020). Poverty, challenging economic conditions, and high levels of English language proficiency are considered the main reasons for this (Brown, 2016). However, several cases in other countries have been reported (Europol, 2020a). The Internet Watch Foundation (2018) found that victims were mostly white females and from relatively wealthy Western backgrounds. This suggests that different forms of LSCSA may exist in different geographical locations although this is not fully reflected in the data. The question is whether LSCSA is occurring primarily in the Philippines or if we are seeing the tip of the iceberg of a globally widespread issue. Although the data in the records provide no clear answer to this question, one can clearly observe that countries such as China, India, Japan, and Brazil recognize the problem and have started their own respective counterprograms. This is also the case for the European Union, which recently released a proposal laying down rules to prevent and combat child sexual abuse, which is also connected to the strategy of a better internet for kids (European Commission, 2022a, 2022b). The proposal has specific definitions for online live abuse including actual and simulated sexually explicit conduct.

The data contained both cases of LSCSA with a facilitator as well as self-generated sexual content featuring children enabled by livestream services. The latter is also found to be rising (Internet Watch Foundation, 2020). In cases of self-generated sexual content featuring children, it is often challenging to evaluate the level of coercion, as some of the young individuals are using streaming services to self-exploit, earn money and receive gifts, or receive “likes” (Internet Watch Foundation, 2018; Jonsson et al., 2014; Terre des Hommes, 2013). In addition, it is not always clear whether a crime is taking place or if (sexual) boundaries are being explored between youth (Koops, 2009). This makes for complex legal challenges as the child can be both considered the offender and the victim (Westlake, 2018). While there is a discussion about the definition of self-generated sexual content featuring children and the extent to which someone is coerced, this group, especially during this digital acceleration, needs more attention, as the data are limited (Internet Watch Foundation, 2018).

Several records found that the age of the offender is between 50 and 60 years (Brown et al., 2020) and while some studies presented a wider range, the average age of the offender was still within that span (Cubitt et al., 2021). This is interesting because, as noted in Brown et al. (2020), a meta-analysis by Babchishin et al. (2011) found that the average age of online sex offenders is significantly younger than the age of the average livestreaming offender found in the records included in this review. This raises the question of why there is a difference between these findings. Since the studies in the scope used law enforcement records and financial transactions, the discovered characteristics describe a convicted offender group. Therefore, it is unclear whether offenders of LSCSA typically are older or older offenders are more likely to get convicted. If it is the latter, it could perhaps have something to do with the type of technology utilized. Convicted offenders of CSEM have been found to use well-established technologies rather than being early adopters of new tools (Steel et al., 2020). Less is known about the facilitator, other than that most of them were family members, female and some had experienced abuse themselves (Napier et al, 2021b). Therefore, more research is needed on both the offender and the facilitator.

The findings on payment methods are inconsistent with current trends. Europol (2021a) states that cryptocurrency, a key payment method on the dark web (Heaslip, 2021), is used to pay for LSCSA. In this respect, a cryptocurrency is considered more secure than bank transfers, and it is challenging to trace the identity of the paying customer. This payment method is undoubtedly a means of staying anonymous on the internet. However, it was found that offenders do not often take privacy measures to hide their identity (International Justice Mission, 2020; Balfe et al, 2015). The data provided by the records found that the most utilized payment services are Western Union and remittance services such as WorldRemit or Paypal (Napier et al., 2021a; Terre des Hommes, 2014; Varrella, 2017). This indicates that newer payment methods such as cryptocurrencies are not utilized or that offenders using these services have not been convicted. It is clear that there is a gap between the data presented by the records and the concerns raised by Europol and other law enforcement agencies about the state of LSCSA “facilitated by new technologies” (Europol, 2021b).

### Psychological Consequences and Trauma

There is limited evidence on specific psychological consequences and possible trauma-related psychopathology specifically for victims of LSCSA. However, Terre des Hommes (2013) found that these victims suffer from high levels of psychosocial distress, which is in line with the findings of studies that have looked at both offline and online child sexual abuse, that is, not specifically livestreaming. Research suggests that the consequences of OCSEA can be as severe and harmful as offline child sexual abuse (Hamilton-Giachritsis et al., 2017; Jonsson et al., 2019; Whittle et al., 2013). A history of childhood abuse increases the chances of developing emotional and behavioral problems, anxiety, depression, and posttraumatic stress disorder. Further, it increases the risk of poor health outcomes throughout the lifespan (Hailes et al., 2019; Murray et al., 2014). Joleby et al. (2020a) found psychological consequences specifically for victims of OCSEA, including psychological suffering, self-harming and suicidal behavior, internalized self-loathing, and impaired relationships. Furthermore, a common theme was self-blaming, among other adverse effects on the health and well-being of the victims in both the short and long term (Joleby et al., 2020b). In addition, research indicates that for victims, knowledge of images of the abuse existing or having been distributed online was related to higher levels of posttraumatic stress symptoms compared to being exposed to child sexual abuse without it being documented (Jonsson & Svedin, 2017).

### Technology

The review found that most of the platforms used by facilitators and offenders are relatively few and seem somewhat outdated: Facebook Live, Skype, and Yahoo Messenger (Napier et al., 2021a; Terre des Hommes, 2013). It is interesting that while technology is developing fast, the findings of a study in 2013 and 2021 have similar outcomes regarding the technical platforms used in LSCSA. Although livestreaming is an established technology and has been so for years (Fecheyr-Lippens, 2010), the functionality has developed in terms of the availability of production devices (cameras), consumption devices (screens), speed, quality (resolution and framerate), stability (no stalling), secure environments, and ease of use. In addition, the COVID-19 pandemic accelerated user knowledge. For comparison, Table A1 in the Supplemental Appendix presents a non-exhaustive overview of some available systems providing livestreaming services today. A key observation is the ease of use of such systems where anyone with any type of device with a camera and microphone, can easily set up a service, and anyone with a simple video app or a vanilla web-browser can receive such streams. Even though many of the livestreaming systems differ slightly in intended use and target user groups, they all provide the same opportunity to set up a livestream easily. Most of the services can be free of charge to a small scale, with a paid option for larger scale setups or higher service guarantees. Nevertheless, the examples show how available today’s technology is to set up both live broadcasts and closed streaming sessions, that is, with the touch of a button. To provide privacy, many of the systems allow private break-out rooms and data (video) encryption, known as end-to-end encryption. It is also possible to be anonymous, both as a producer, making the stream available, and as a consumer, viewing the stream. Alternatively, offenders may use modern cloud services to set up their own video streaming servers. For example, the Amazon Interactive Video Service, Azure Media Services, Google Cloud Video Intelligence Streaming API, or IBM Watson media streaming platform can be used to encode and stream video at large scales, which would be ideal for creating interactive video. Furthermore, as shown in Table A1 in the Supplemental Appendix, the various systems have strict requirements regarding the allowed content. Almost all forbid nudity, adult content, pornography, and any type of child abuse, in particular child sexual abuse material. Most systems also have a lower age limit (typically at least 13 years) to prevent minors from using the service. The terms also typically state that any attempt to break these rules will result in a report sent to law enforcement and the National Centre for Missing and Exploited Children. However, given the various technical properties of the systems, it is hard to detect these illegal streams. Some systems mention that they have automatic surveillance, for example using Artificial Intelligence, to detect illegal content, but this is immature, and the video content may also be “unreadable” due to encryption. Many details are missing on how automatic detection is done and its possibilities. For example, it is not clear whether the automatically detected content is verified by a human observer. Overall, providing such services for “private events” without being detected is relatively easy. The platforms are not technologically sophisticated and require no specialized technical knowledge for users. Research has found that offenders use both older and newer technologies for online child sexual abuse (Steel et al., 2020). However, the findings of the studies in this review present only the former. Considering the accessibility of different systems and how easy it is to create one’s own stream, it is surprising that none of the records in the scope have detected a wider range of systems used.

During the COVID-19 pandemic, internet usage increased by 50% in several European countries (da Silva et al., 2021). Europol (2020a) found there has been “a significant increase in activity relating to child sexual abuse on both the surface web and dark web during the COVID-19 lockdown period” (p. 3). For example, 6 months after the first lockdown in England, a 17% increase in online sexual crimes against children was recorded (Harris et al., 2021). Video streaming, in general, and the use of livestreaming services have risen (da Silva et al., 2021; Statista, 2022). Furthermore, conference calls or video conferencing has become the new norm, using the platforms presented in Table A1 in the Supplemental Appendix such as Zoom, Teams, and Skype. In addition to accessibility and user knowledge, the use of home offices has increased. This environment could provide more privacy and therefore increase the risk of work equipment being used to download or consume CSEM (Netclean, 2020). This increased knowledge and availability could lead to a further increase in the LSCSA in the future. The fact that the abuse is streamed in real time also presents a possibility for the child to be found and rescued which is why more research is needed to further understand LSCSA and develop measures to protect and prosecute.

## Conclusion

The review confirmed that there is a limited body of research that has examined this issue of LSCSA. Most of the data stems from the Philippines and is based on a combination of case reports and law enforcement data. Differences in terminology, study design, and inclusion criteria of the population studied present a challenge to drawing general conclusions on the current state of LSCSA. There is no legal definition for LSCSA, and it is not considered a stand-alone crime creating challenges for criminalization and victim protection. There are limited studies on the psychological consequences for victims. The records indicate that trusted platforms are used to contact the victim or facilitator and view the livestream. Considering the plethora of livestreaming services available today, the records appear to underrepresent this digital development. Similarly, the records point to only a handful of well-established services for payment methods, and do not reveal information on emerging financial technologies such as cryptocurrencies. The average age of a live-streaming offender was found to be older than the average age of online child sexual abuse offenders. Therefore, it is unclear whether the findings represent the global population of livestream offenders or if other factors drive this age difference, such as unfamiliarity with more secure streaming technologies among the older population. In addition, privacy considerations pose a challenge to investigation and detection by law enforcement. This field is in the beginning stages of gathering data, creating knowledge, and understanding the implications for victims and society. It is clear that the available data only provides a small window into a potentially much larger issue.

## Limitations

Although this scoping review was conducted through a systematic process to ensure transparency and rigor, it has several limitations. There is a possibility that the review may have missed other relevant studies. This can have several causes such as the database selection, the definitions used in the searches, and the exclusion of studies published in languages other than Danish, English, Norwegian, and Swedish. In addition, because of the inclusion of grey literature, which was often found through scanning references, it is possible that significant reports were overlooked. Further, due to the nature of a scoping review, a formal assessment of methodology and a quality assessment of the included reports were not performed. However, it must be noted that a formal assessment falls outside of the scoping review’s scope and focus.

## Recommendations

It is essential for practitioners to be aware of the potential online components in child sexual abuse cases and the increasing prevalence of LSCSA. LSCSA can lead to revictimization when the material (CSEM) is spread online. Research into the psychological consequences and trauma and the effects on disclosure is needed to establish the specific needs and care for victims of LSCSA. It is important this research takes gender and cultural dimensions into account.

Policymakers must be made aware of the rising threat livestreaming services present to society and its children. Policymakers should focus on holding companies accountable for the platforms they provide. What would help in this matter is to create a legal framework and definition for LSCSA and review both international and national legal frameworks so that offenders can be convicted, and the protection and care of the victim established.

There is a clear lack of research on LSCSA, and much of the data stems from cases from the Philippines. Therefore, it would be beneficial to look at other geographical locations to study the prevalence of LSCSA. In addition, it is important to gain a better understanding of the different types of OCSEA facilitated through livestreaming services. In particular, the category of self-generated sexual content featuring children and adolescents is an area where more research is needed. It is argued that a great deal of research on CSEM is done through retrospective examination, and although valuable insights are provided, little new knowledge is added to assist law enforcement adequately (Westlake, 2020). This review confirms that the data on LSCSA, with some exceptions, fall in that same category. Even though the technologies used in the cases examined might still be utilized today to some degree, something is likely being missed. It is therefore essential that future research focus on that gap through scientific work. For example, several providers state that they use machine learning (a type of artificial intelligence) to detect anomalies, but the models are still in their infancy, and more work is needed at the same time as access to the data is required (i.e., with all the challenges this will infer). Moreover, interdisciplinary collaborations (law enforcement, the scientific community, tech companies, and children themselves) are at the core of the types of research needed to understand the LSCSA and its developing methods in the future. With the digital acceleration that the COVID-19 pandemic has brought upon the world, we must do all we can to protect children from harm and to ensure their digital rights, both online and offline (United Nations, 2021). The implications for practice, policy, and research are presented in Table 4.

**Table 4.** Implications for Practice, Policy, and Future Research.

	Implications and Recommendations
Practice	* Practitioners need to be aware of the online components in child sexual abuse cases. *Inclusive and gender-sensitive research of the psychological consequences and trauma and the effect on disclosure is needed to adequately meet the specific needs and care for victims of LSCSA.
Policy	* Policymakers must be made aware of the rising threat livestreaming services present. * Policymakers should work on making companies accountable for the platforms they provide. * Create a legal framework and definition for LSCSA. * Review both international and national legal frameworks. *Facilitate interdisciplinary collaborations.
Future research	* Focus on other geographical locations with high poverty rates, access to technology and high English proficiency levels to study the prevalence of livestreaming of child sexual abuse. * Focus research on self-generated sexual content featuring children and adolescents. * Focus research on technical trends within livestreaming services to understand the prevalence of LSCSA.

*Note*. LSCSA = livestreaming of child sexual abuse.

## Supplemental Material

sj-docx-1-tva-10.1177_15248380221147564 – Supplemental material for Livestreaming Technology and Online Child Sexual Exploitation and Abuse: A Scoping Review Click here for additional data file. Supplemental material, sj-docx-1-tva-10.1177_15248380221147564 for Livestreaming Technology and Online Child Sexual Exploitation and Abuse: A Scoping Review by Catharina Drejer, Michael A. Riegler, Pål Halvorsen, Miriam S. Johnson and Gunn Astrid Baugerud in Trauma, Violence, & Abuse

## Notes

1. UN Convention on the Rights of the Child, the Optional Protocol to the CRC on the Sale of Children, Child Prostitution, and Child Pornography, the EU Directive 2011/93/EU on combating sexual abuse, sexual exploitation, and child pornography, the Council of Europe Convention for the Protection of Children against Sexual Exploitation and Sexual Abuse No. 201 (Lanzarote Convention), and the Convention on Cybercrime No. 185 (Budapest Convention), and the International Labour Organisation Convention No. 182 on the Worst Forms of Child Labour.
