# Supplementary material for: Livestreaming Technology and Online Child Sexual Exploitation and Abuse: A Scoping Review
Source: Trauma Violence Abuse. 2023 Feb 2;25(1):260–74. doi: 10.1177/15248380221147564 (PMC10666494; doi:10.1177/15248380221147564)
Supplement: sj-docx-1-tva-10.1177_15248380221147564 – Supplemental material for Livestreaming Technology and Online Child Sexual Exploitation and Abuse: A Scoping Review [file sj-docx-1-tva-10.1177_15248380221147564.docx]

# APPENDIX

**Table A1.** *Overview of live streaming systems available today (non-exhaustive)**

| **Free** | **Anonymous producer** | **Anonymous viewer** | **Reports abuse to NCMEC** | **Excerpts from the term sheet and policies** | **References** |
| --- | --- | --- | --- | --- | --- |
| Skype | | | | | |
| *F* | *AP* | *AV* | *Re.* | *Excerpts from the term sheet and policies* | *References* |
| Y | Y | Y | Y | 3.a.II - ii. no activity that exploits, harms, or threatens to harm children.  3.a.iv - no nudity, pornography, violence, … Microsoft leverages a variety of tools to detect CSEAI, including hash-matching technology (e.g., PhotoDNA) and other forms of proactive detection. Microsoft has also made available in-product reporting for products such as OneDrive, Skype, Xbox, and Bing, whereby users can report suspected CSEAI or other violating content. .. Microsoft reports all apparent CSEAI to NCMEC | [https://www.skype.com/en/free-conference-call/](about:blank)  [https://www.microsoft.com/en-us/servicesagreement/](about:blank)  [https://www.microsoft.com/en-us/corporate-responsibility/digital-safety-content-report?activetab=pivot_1%3aprimaryr3](about:blank) |
| Facebook live | | | | | |
| *F* | *AP* | *AV* | *Re.* | *Excerpts from the term sheet and policies* | *References* |
| Y | N | Y | Y | We do not allow content that sexually exploits or endangers children. When we become aware of apparent child exploitation, we report it to the National Center for Missing and Exploited Children (NCMEC), in compliance with applicable law. | [https://www.facebook.com/formedia/tools/facebook-live](about:blank)   [https://transparency.fb.com/nb-no/policies/community-standards/child-sexual-exploitation-abuse-nudity/](about:blank) |
| YouTube | | | | | |
| *F* | *AP* | *AV* | *Re.* | *Excerpts from the term sheet and policies* | *References* |
| Y | N | Y | Y | Don’t post content on YouTube if it fits any of the descriptions below.  **Sexualization of minors**: Sexually explicit content featuring minors and content that sexually exploits minors.  We report content containing child sexual abuse imagery to the NCMEC, who work with global law enforcement agencies. | [https://support.google.com/youtube/answer/2474026?hl=en](about:blank)  [https://support.google.com/youtube/answer/2801999?hl=en&ref_topic=9282679](about:blank) |
| Snapchat | | | | | |
| *F* | *AP* | *AV* | *Re.* | *Excerpts from the term sheet and policies* | *References* |
| Y | N | N | ? - report to authorities | Snap Inc. Terms of Service  Seems to just say that you cannot use the service if you:  “you are not a convicted sex offender; and”  but below this you find the **Community Guidelines:**  Sexually Explicit Content  - We prohibit accounts that promote or distribute pornographic content.  - We report all instances of child sexual exploitation to authorities. Never post, save, or send nude or sexually explicit content involving anyone under the age of 18 — even of yourself. Never ask a minor to send sexually explicit content. | [https://www.snapchat.com/add/live.oficial](about:blank)  [https://snap.com/en-US/community-guidelines](about:blank)  [https://filme.imyfone.com/video-editing-tips/do-live-video-on-snapchat/](about:blank) |
| Kik | | | | | |
| *F* | *AP* | *AV* | *Re.* | *Excerpts from the term sheet and policies* | *References* |
| Y | Y, but logs | Y, but logs | ? | What’s Not Permitted?  Material that is unlawful, obscene, defamatory, libellous, threatening, pornographic, harassing, hateful, racially or ethnically offensive, or encourages conduct that would be considered a criminal offense, give rise to civil liability, violate any law, or is otherwise inappropriate as determined in our sole discretion; | [https://en.wikipedia.org/wiki/Kik_Messenger](about:blank)  [https://www.kik.com/terms-of-service/](about:blank) |
| Omegle / OmeTV | | | | | |
| *F* | *AP* | *AV* | *Re.* | *Excerpts from the term sheet and policies* | *References* |
| Y | Y | Y | ? | It is forbidden to:  - Appear naked or in underwear.  - Suggest ‘virtual sex’ to interlocutors.  If you see a child in the video chat, please send a complaint to our moderators, so they can quickly respond and ban the minor from using the service. | [https://ome.tv](about:blank)  [https://ome.tv/rules/](about:blank) |
| WhatsApp | | | | | |
| *F* | *AP* | *AV* | *Re.* | *Excerpts from the term sheet and policies* | *References* |
| N? | N | N | Y | Does not say much about sex/children/etc. BUT  ‘If we detect apparent CSAM, we report it to the NCMEC.’ | [https://www.whatsapp.com/features](about:blank)  [https://www.whatsapp.com/security](about:blank)  [https://www.whatsapp.com/legal/](about:blank)  [https://faq.whatsapp.com/general/how-whatsapp-helps-fight-child-exploitation](about:blank)  [https://www.liveme.com/app/livemepay/dist/uspay.html?from=pc#/](about:blank#/) |
| TikTok | | | | | |
| *F* | *AP* | *AV* | *Re.* | *Excerpts from the term sheet and policies* | *References* |
| Y | N | Y | Y | You may not:  - promote sexually explicit material  In certain circumstances, TikTok is subject to legal obligations to report certain user data either to law enforcement authorities or to designated bodies such as the U.S. National Centre for Missing and Exploited Children (‘NCMEC’). | [https://www.tiktok.com/legal/terms-of-service?lang=en](about:blank)  [https://www.tiktok.com/legal/law-enforcement?lang=en](about:blank) |
| LiveMe | | | | | |
| *F* | *AP* | *AV* | *Re.* | *Excerpts from the term sheet and policies* | *References* |
| Y | N | Y? | ? | Sexually suggestive behaviours and content or activities with sexual innuendo are not allowed.  LiveMe introduces a total ban on use by underage users (18).  one or more improperly dressed children are shown in the live stream without the company of an adult, or the live stream pictures can adversely affect children's psychological and/or emotional states, are deemed in violation of the child safety rules. | [https://www.liveme.com](about:blank)  [https://www.liveme.com/app/protocol/dist/communityPolicy.html](about:blank)  [https://apps.apple.com/us/app/liveme-live-stream-go-live/id1089836344](about:blank) |
| Instagram | | | | | |
| *F* | *AP* | *AV* | *Re.* | *Excerpts from the term sheet and policies* | *References* |
| Y | N | N? | Y | You must not be a convicted sex offender.  we don’t allow nudity on Instagram.   Offering sexual services, … are also not allowed.  We have zero tolerance when it comes to sharing sexual content involving minors.   We report all apparent child pornography to NCMEC | [https://help.instagram.com/292478487812558](about:blank)   [https://help.instagram.com/581066165581870](about:blank)   [https://help.instagram.com/477434105621119/?helpref=hc_fnav](about:blank) |
| Zoom | | | | | |
| *F* | *AP* | *AV* | *Re.* | *Excerpts from the term sheet and policies* | *References* |
| Y | N | Y | Y | We process certain personal data in order to protect vital interests for the purpose of detecting and preventing illicit activities that impact vital interests and public safety, including child sexual abuse material;  We permanently suspend accounts that we determine have transmitted, displayed, stored, shared, or promoted CSAM on our platform. Additionally, we alert NCMEC. We may also notify law enforcement and other entities as required by law. | [https://explore.zoom.us/en/privacy/](about:blank)   [https://explore.zoom.us/en/csam/](about:blank) |
| Teams | | | | | |
| *F* | *AP* | *AV* | *Re.* | *Excerpts from the term sheet and policies* | *References* |
| Y * | N | Y | Y | Don’t engage in any activity that exploits, harms, or threatens to harm children.  We leverage a variety of tools to detect CSEAI, including hash-matching technology (e.g., PhotoDNA) and other forms of proactive detection. Microsoft has also made available in-product reporting whereby users can report suspected CSEAI or other violating content.  Microsoft removes content that contains apparent CSEAI. As a US-based company, Microsoft reports all apparent CSEAI to the National Centre for Missing and Exploited Children (NCMEC) via the CyberTipline, as required by US law.  * free participants, but must have a user to make a video session. | [https://www.microsoft.com/en-us/corporate-responsibility/digital-safety-content-report?activetab=pivot_1%3aprimaryr3](about:blank)  [https://aadcdn.msauth.net/ests/2.1.12470.11/content/agreements/tou/en-us/tou.txt](about:blank) |
| Google Meet | | | | | |
| *F* | *AP* | *AV* | *Re.* | *Excerpts from the term sheet and policies* | *References* |
| Y | N | Y | Y | Do not use Meet to create, upload, or distribute content that exploits or abuses children. This includes all child sexual abuse materials.  We will take appropriate action, which may include reporting to the National Center for Missing & Exploited Children, limiting access to product features, and disabling accounts. | [https://apps.google.com/meet/](about:blank)  [https://support.google.com/legal/troubleshooter/1114905#ts=1115658%2C1115686](about:blank#ts=1115658%2C1115686)  [https://support.google.com/meet/answer/9847091?hl=en](about:blank) |
| Twitch | | | | | |
| *F* | *AP* | *AV* | *Re.* | *Excerpts from the term sheet and policies* | *References* |
| Y | N | ? * | Y | Nudity and sexually explicit content or activities, such as pornography, sexual acts or intercourse, and sexual services, are prohibited.  Child exploitation will be reported to authorities via the NCMEC.  *can view videos from the front page without being logged in | [https://www.twitch.tv/p/en/legal/terms-of-service/](about:blank)  [https://safety.twitch.tv/s/article/Community-Guidelines?language=en_US](about:blank) |
| Slack | | | | | |
| *F* | *AP* | *AV* | *Re.* | *Excerpts from the term sheet and policies* | *References* |
| Y | N | N | ? | Does not explicitly seem to mention ‘CSAM’ issues. | [https://slack.com/video-conferencing](about:blank)  [https://slack.com/legal](about:blank) |
| Discord | | | | | |
| *F* | *AP* | *AV* | *Re.* | *Excerpts from the term sheet and policies* | *References* |
| N | N | N | Y | Here are some rules for content on Discord: e.g.,  -You must apply the NSFW label to channels if there is adult content in that channel.  -You may not sexualize minors in any way.   - You may not share sexually explicit content of other people without their consent  Reports abuse to NCMEC | [https://discord.com/nitro](about:blank)  [https://discord.com/guidelines](about:blank)  [https://discord.com/terms](about:blank) |

* Other similar examples include LinkedIn Live, Periscope, Unscreen, Reddit Live, Panopto, Wowza, Brightcove, Vimeo, Dacast, Kaltura, and StreamShark.
